# Emergency Management of Tension Pneumothorax for Health Professionals on Remote Cat Island Bahamas

**DOI:** 10.7759/cureus.1390

**Published:** 2017-06-25

**Authors:** Tia Renouf, Michael Parsons, Leathe Francis, Cristian Senoro, Caroline Chriswell, Rose Saunders, Charles Hollander

**Affiliations:** 1 Emergency Medicine, Memorial University of Newfoundland; 2 Bahamas Health Board; 3 Nursing, Orange Creek Clinic; 4 Trained Clinical Nurse, Smith's Bay Clinic; 5 Volunteer, Old Bight Clinic

**Keywords:** rural medicine, simulation, cat island, pneumothorax

## Abstract

Patients living in remote areas have higher rates of injury-related death than those living in cities. Rural and remote health professionals working in sparsely populated places, such as Cat Island Bahamas, may have scant resources for treating emergency conditions. Local health professionals must be prepared to rely solely upon clinical judgment to perform emergency “high-stakes low-frequency” procedures while also accurately and effectively communicating with distantly located receiving specialists. However, these health providers may not recently have performed or had the opportunity to practice such emergency procedures. Telesimulation may be a useful way to teach remote practitioners both emergency procedures and communication skills. This technical report describes a simulation exercise for teaching these skills.

## Introduction

Patients living in remote areas of the world have higher rates of injury-related death than those living in cities [1]. However, health professionals working in remote locations often have little opportunity to practice “high-stakes low-frequency” procedures. Furthermore, remote health professionals’ critically ill or injured patients must be stabilized and transported to a referral centre. Weather may delay this process for long periods. This characterizes the remote medicine context, seldom experienced and, therefore, sometimes poorly understood by receiving specialists working at referral centres. A contextual misunderstanding between rural/remote health professionals and urban consultants can produce miscommunication between the two groups and potentially affect patient safety [2].  

The Specificity of Practice Theory suggests that it is ideal to practice a skill in the setting where it will be performed, as context drives action [3]. For example, the urban emergency room might be a suboptimal setting to teach a procedure, such as a needle thoracostomy, to remote practitioners whose context fundamentally differs with respect to available medical staff and instruments. A more effective approach may be to teach needle thoracostomy in-situ, in this case, in the remote clinic itself [4].

Skilled educators are necessary for successful training anywhere [5]. This presents a gap for rural and remote practitioners because trained educators generally work in academic centres, from which it is impractical and costly to be transported away to a remote island. Telesimulation, “a process by which telecommunication and simulation resources are utilized to provide education, training and/or assessment to learners at an offsite location” [6], may help to overcome distance-related barriers and close this gap.

Tension pneumothorax (TPX) is a life-threatening condition that must be recognized immediately and treated urgently before any investigations are performed. While TPX generally occurs in traumatic and critical care settings, a spontaneous pneumothorax may also come under tension. TPX is treated with a needle thoracostomy. Complications of needle thoracostomy are few but include hemorrhage, vascular injury, and ineffective catheter placement [7]. A chest tube must be placed before the patient is evacuated by air. Telesimulation, the subject of this report, may be a valuable way to teach to remotely placed practitioners both a high-stakes low-frequency skill (needle thoracostomy) and necessary communication skills.

Telesimulation, in this case, connects the learners (all graduate health professionals at a clinic on Cat Island, Bahamas) to mentors at an academic centre at Memorial University of Newfoundland, Canada. The simulation could be applied to any remote setting and academic centre. Cat Island’s 1,522 inhabitants live in several communities dotting an approximately 50 mile-long coast [8]. Five nurses and one physician work from three separate clinics. The nurses provide front-line care on Cat Island and, therefore, must be prepared both to treat high-stakes low-frequency conditions and (along with physicians) to communicate their contexts to the distantly placed urban specialists with whom they consult.

The learning objectives for this case are:

1. Clinically recognize tension pneumothorax;

2. Perform needle thoracostomy and recognize needle thoracostomy malfunction;

3. Communicate for patient referral/transport.

## Technical report

**Figure 1:**
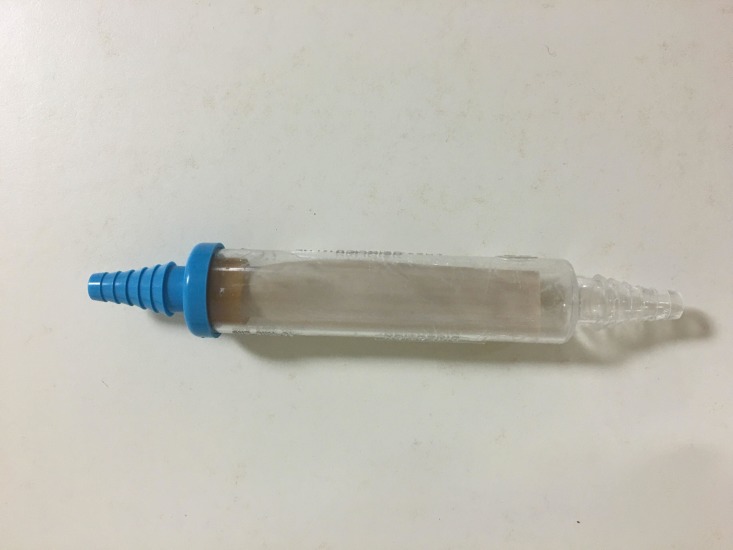
Flutter valve, attach to tubing and angiocatheter for treatment of TPX TPX: tension pneumothorax

This scenario is organized according to the context, input, process, and product (CIPP) evaluation checklist model [9]. Table 1 details the context of this scenario and lists all the necessary inputs, including a commercial flutter valve as shown in Figure 1. 

**Table 1:** Context and Inputs for the Simulation Scenario TPX: tension pneumothorax

CONTEXT
This simulation is performed in a small rural clinic on Cat Island. The learners are postgraduate nurses and physicians. A practice session or “dry run” will consider time zone differences between Cat Island and Newfoundland (1.5 hours). The scenario begins with a sole health provider (in this case, a nurse) who must clinically diagnose TPX, call for the physician, and treat the TPX immediately. When the physician arrives, he or she works with the local physician to establish communication with the accepting physician consultant.
INPUTS
Personnel
Two learners (one nurse and one physician) at the Cat Island clinic If available, one confederate with abnormal anatomy: tracheal deviation Two mentors, one simulation centre staff (facilitator), one technical support person, and one confederate at the Memorial University site. The confederate will act as the receiving specialist physician.
Simulation Set-up
Locally made TPX task trainer This task trainer uses an inflated surgical glove that represents a TPX, and is placed beneath a set of pork ribs. 2 x 14 gauge long (at 3 cm) angiocatheters Tape Oxygen and tubing Oxygen saturation monitor Cardiac monitor Intravenous fluid and tubing 3-way stopcock to aspirate pneumothorax One finger of a surgical glove, cut and removed (or commercial flutter valve) Telephone or radio to simulate communication with off-island physician
Technology Set-up
Computers configured with group SKYPE ®, VSEE® or other technology Microphones at both sites Headsets at both sites External computer speakers at both sites. 1-2 external camera(s) connected to Cat Island clinic computer 2 cameras at Memorial University: (1) static computer camera that allows eye contact and (2) mentor head-mounted camera to allow hands-free

### Process

Roles should be assigned in advance. The leader in this simulation (a nurse) is told that a young, tall, thin male has arrived at the clinic in acute respiratory distress. The leader will be asked by the facilitator to state what medical problems they anticipate. The team will begin the simulation when ready. Two online mentors at Memorial University will (1) assign roles, provide information, and ensure adherence to the scenario and (2) observe the simulation and record information for the debriefing. A confederate at Memorial University will act as the accepting consultant physician. Technical support will be available.

The correct action in this scenario would be for learners to recognize a tension pneumothorax and the need to immediately place a needle thoracostomy tube. If the leader does not place this tube, the facilitator should ask him or her to justify this action, as the procedure is strongly indicated. Furthermore, if the leader continues to defer tube placement, the team should be informed that the patient deteriorates and is exhibiting worsening respiratory distress and vital signs. If this does not prompt tube placement, the patient will go into pulseless electrical activity (PEA) arrest and the facilitator of the scenario should instruct the team leader to place the tube immediately. This action results in patient improvement and normalization of vital signs. If the patient deteriorates again, TPX reaccumulation is diagnosed, and the learner will place a second needle thoracostomy tube on the same side as the first tube. This action will result in patient improvement and vital signs will again return to normal.

When a second learner (physician) arrives, a call is placed to the receiving specialist at a tertiary care centre. Recognizing a chest tube will have to be placed prior to air transport, the receiving specialist will ask for the physician on Cat Island to do so. The Cat Island physician will relate that team members are not comfortable placing a chest tube. Nobody has performed the procedure recently, and the clinic does not seem to have all available supplies. They will relate that the TPX has resolved, the patient is being monitored and is stable, and that a medical evacuation team member can place the chest tube and bring to Cat Island the necessary equipment.

The scenario ends when the patient has a functional needle thoracostomy tube, and transfer plans are in place. If transportation is not immediately available, plans for continuing monitoring are verbalized.

A feedback/debriefing session is initiated and the reviewer asks open-ended questions to evoke reflection. Learning objectives are reviewed during the debriefing, and new knowledge is consolidated. All feedback should be constructive and respectful. 

### Products/outcomes

The nurse must correctly place the first thoracostomy tube and then place a second one when it becomes obstructed. Along with the nurse, the physician must consider with the team whether to place a chest tube in the clinic or wait for the incoming medical evacuation (medevac) team to do so. He or she must then communicate effectively their local context and capabilities to the receiving consultant, along with a list of necessary equipment they must bring.

### Case

This is a case of a left-sided spontaneous tension pneumothorax (TPX) in a young male. He presents with acute chest pain and shortness of breath, hypoxia, hypotension, tachypnea, and tachycardia. Breath sounds are absent on the left, his trachea has deviated to the right, and his jugular veins are distended. TPX is a purely clinical diagnosis that must be made urgently and treated immediately with a needle thoracostomy. Continued monitoring is required as these cannulae often become blocked, causing the TPX to re-accumulate. Consideration must be made for chest tube placement and communication must be initiated with a remotely placed consultant. See Table 2 for the detailed scenario template.

**Table 2:** The Detailed Scenario Template, which Guides the Scenario and Outlines Necessary Materials for the Case

Pre-Scenario		
You are working in a remote island clinic. An 18-year-old male comes to the clinic acutely short of breath. He has a sore chest. The nearest hospital is 30 minutes by air and the nearest doctor is 30 minutes away by road.		
History (Hx)		
Allergies	none	
Medications	Salbutamol as needed	
Past Medical Hx	Asthma (mild)	
Initial Vitals	Temperature (T) 36.5 (axillary) // heart rate (HR) 144 (sinus) // blood pressure (BP) 100/65 // respiratory rate (RR) 32 // oxygen saturation (SpO2) 80% room air (RA) The patient is alert, anxious, in pain, and very short of breath.	
General appearance	6’3” and very thin, anxious appearance. Pale and diaphoretic with signs of respiratory distress.	
Central Nervous System (CNS)	Nil	
Chest	Heart sounds normal, breath sounds absent on the left, trachea deviated to the right. Percussion note is hyper-resonant on the left.	
Abdomen	Soft; non-tender	
Extremities	Unremarkable	
Learning Objective 1: Clinically recognize tension pneumothorax		
Initial Assessment/Stabilization		
General assessment	Vitals signs and clinical diagnosis	Expected Action
Establish clinical diagnosis of tension pneumothorax using Airway, Breathing, Circulation (ABC) approach	Airway (A) – airway patent and protected Breathing (B) – RR 32, respiratory distress present, trachea deviated to right, absent breath sounds on left with increased tympany to percussion Circulation (C) – BP 100/65, HR 144, SAO2 92, heart sounds normal, jugular venous pressure (JVP) elevated	Immediately perform needle thoracostomy in the left anterior second rib space mid-clavicular line, placing 14 or 16 gauge angiocatheter above the top of the second rib and attaching flutter valve or connecting cannula from the chest with a stopcock and intravenous (IV) tubing to underwater seal.
If practitioner waits for doctor to arrive	BP 80, HR 150, SAO2 88, patient now very distressed.	Immediately perform needle thoracostomy.
If no needle thoracostomy placed, prompt the learner that the patient looks worse and suggest intervention.	BP now 70, HR 188, SAO2 80.	If no intervention, patient unconscious, no pulse: pulseless electrical activity (PEA) arrest.
Learning Objective 2: Perform needle thoracostomy		
Perform needle thoracostomy	Vitals stable	Correct placement is left mid-clavicular line above 2^nd^ rib. Connect to flutter valve as above. If there is no flutter valve, one may cut off one finger of a surgical glove and affix that instead.
Place large bore long angio-catheter in left 2^nd^ intercostal space above rib	Vitals normalize	Place patient on cardiac and oxygen (O2) sat monitors if available. Obtain IV access. Consider analgesia. Doctor arrives. Gather chest tube equipment. Make contact with hospital.
Patient becomes unstable.	RR 44, BP 110/60, Pulse (P) 140. Respiratory distress.	Reassess the patient and place a second angiocatheter in the left anterior chest. Prepare for chest tube placement if doctor has arrived.
Learning Objective 3: Prepare for transport		
The patient must be transported by air off the island. Consider whether chest tube is to be placed on the island or patient is able to wait for transport team. Must occur before the patient is transported by air.	Vitals stable	Call surgeon and arrange transport to the trauma centre. Consider having a unit of packed red blood cells available during transport.
Contact is made with remote off island medevac team	Communicate that patient is stable, TPX has been treated with a large bore angiocatheter in the anterior chest.	Maintain monitors, have chest tube equipment ready at the bedside. Ensure oxygen and reevaluate need for analgesia.
If remote consultant requests chest tube placement at remote island clinic, team must come to the consensus as to whether they are comfortable doing this procedure.	Communicate to distant consultant, the local context of the remote island clinic, and discuss risks and benefits of performing the chest tube procedure.	Advise medevac team of necessary drugs or equipment they must bring (i.e., not available at the Cat Island clinic).

### Pre-briefing

Before starting the case a formal pre-briefing session is held with all learners using distance technology, such as group Skype® (Skype, Inc., Redmond, WA), VSee® (VSee, Sunnyvale, CA), or another low-cost option that allows multiple visual participants. The pre-briefing marks the beginning of the simulation, establishes a safe learning environment, and introduces the fiction contract. Limitations of the simulation are addressed. In this case, particular attention is paid to potential technical issues with the telesimulation setup. Learners are advised that the session is strictly formative.

Prior to the session, team members at Memorial University familiarize themselves with a detailed scenario template. Two mentors and a confederate (the consultant) are present from the Memorial University site. One mentor runs the scenario while the other observes and prepares for the subsequent debriefing. Technology support should be available.

### Technological considerations

Cat Island has limited infrastructure. Clinics have an established but unpredictably reliable internet connection. Clinic staff are familiar with Skype® technology, which may be enhanced inexpensively in order to simultaneously show multiple participants. Although clinic space is limited, strategically placed high-definition cameras showing multiple perspectives may enhance preceptor visualization. More cameras may, however, reduce the telecommunication quality. This may be overcome using an established mainframe at Memorial University. One must be mindful of time zone differences and also brief delays sending and receiving sounds between sites. It may be more effective to use hand signals (thumbs up, thumbs down) rather than to verbally interrupt the scenario. If used, hand signals must be slow and clear. Prior to the session, a dry run will familiarize all users with the technology and help minimize problems that may arise during the scenario. Additionally, there should be a plan to reconnect in the event of an unexpected interruption. Table 3 provides tips for using telesimulation.

**Table 3:** Tips for Using Telesimulation

Tips for Using Telesimulation
Consider time zone differences as they relate to teacher and learner schedules Ensure clinic and equipment are free Ensure learners are free from clinical/administrative duties Be prepared for brief delays in audio transmission between sites. Consider using (slow, deliberate) hand signals instead Ensure teachers have adequate views of simulation from one or more high definition cameras placed in clinic Consider using both static and head-mounted for mentors at Memorial University If available, use external microphones, speakers, and headsets Have a call back plan/tech support in case of unexpected interruption Have a dry run to address potential technological challenges, including software concerns, such as password access and connection to Memorial University mainframe computer system Learn and use participants’ names and address them in a collegial manner Use an empathetic tone and establish eye contact Establish a “sense of alliance” between teachers and learners during prebriefing Deconstruct technical and communication skills into observable behaviours during debriefing Be attuned to learner vulnerability, given the absence of emotional nuances and cues online

### Feedback and debriefing

Most learning in simulation-based medical education occurs during the debriefing [5]. Tele-debriefing may be undertaken with a variety of video conferencing platforms. Learners in one study of web-based simulation, however, preferred in-person debriefing because they perceived that distance technology impaired learner-faculty communication [10]. Studies suggest that both undergraduate [11] and graduate [12] learners accept face-to-face feedback more readily if they have a relationship or “sense of alliance” with their teachers. It may be challenging to create the visual and auditory cues that comprise this sense of alliance via long distance. The prebriefing, done before the simulation, may help to do so by introducing mentors and learners. The presence of a coach or a supportive peer group can aid face-to-face feedback on communications skills, in part by making the learner’s “self” epiphenomenal to the communications process. This allows communication to be deconstructed and framed as a series of modifiable behaviours, allowing the learner to reach achievable goals [13-14]. The same may apply to tele-debriefing.

### Post-scenario didactics

A didactic session held after the debriefing will help instructors to illuminate knowledge gaps and cement new learning. 

Learning Objective 1: Clinical Recognition of Tension Pneumothorax

Tension pneumothorax is a life-threatening condition that must be recognized immediately and treated urgently and accurately. While it generally occurs in traumatic and critical care settings, a spontaneous pneumothorax may also come under tension. Air in a TPX increases within the pleural cavity each time the patient inhales, but air cannot escape during exhalation. As a result, the pneumothorax comes under tension and progressively worsens with each subsequent breath. Air in an open or simple pneumothorax is able to escape during exhalation, so tension does not develop. TPX is diagnosed by noting significant respiratory distress and tracheal deviation away from the affected side, along with absent breath sounds and a hyper-resonant percussion note on the affected side. Patients may be significantly hypotensive and tachycardic and, if left untreated, may deteriorate into pulseless electrical activity arrest.

Learning Objective 2: Performance of Needle Thoracostomy and Maintenance of Needle Thoracostomy Function

When TPX is recognized, a long large-bore angiocatheter must be placed in the second intercostal space (above the rib, to avoid the neurovascular bundle on the inferior aspect of the rib), at the mid-clavicular line of the affected side. Alternatively, one may use the fifth intercostal space in the anterior or mid-axillary line. The diaphragm generally does not move above the fifth intercostal space, and placing the angiocatheter here avoids the pectoralis major muscle. While the sternomanubrial joint provides a landmark for the second rib, one may simply use the highest easily palpable rib space in the axilla. The cut finger of a surgical glove functions like a flutter valve when fixed to the end of the angiocatheter, and allows air to escape during patient exhalation. 

Needle thoracostomies often become blocked with tissue and fluid, which is why a large-bore cannula is used. If the cannula does block, the TPX may reoccur. In order to recognize this, the patient must be observed and maintained on cardiac and oxygen saturation monitors. Recurrent TPX is treated with a second needle thoracostomy tube, placed alongside the original. Consideration should be given to aspirating the pneumothorax with a syringe and stopcock or placing a chest tube.

Learning Objective 3: Communication with Consultants for Patient Air Transport

The Cat Island physician must contact the off-island consultant who will accept the patient in transfer. If the consultant recommends that a chest tube be placed at the clinic, the clinic health professionals must decide as a group whether they are comfortable performing this procedure. It is important to communicate to the consultant that the TPX has been decompressed (and if the pneumothorax has been aspirated) and that the patient is now stable. The Cat Island physician must also communicate the clinic context and staff capabilities, in addition to requesting necessary equipment that may not be in stock.

## Discussion

Distance technologies have been used to transfer knowledge and skills in a number of rural and remote settings [15-16]. Telesimulation has been used to teach a variety of technical emergency medicine skills, such as surgical knots [17] and intraosseous needles [18]. It has also been used to teach resuscitations that require team communication skills [19-20]. However, little has been reported on its utility for teaching both (a) a high-stakes low-frequency procedure and (b) communication skills between rural health practitioners and distantly-placed urban consultants.

This telesimulation scenario connects experienced interdisciplinary health professionals at a clinic on the remote Cat Island, Bahamas to mentors at Memorial University of Newfoundland, Canada. It teaches both procedural and communication skills. Communication skills are needed to transfer information between physicians working in different contexts. The patient in this case, for example, will need to have a chest tube placed before evacuation by air from Cat Island to the referral centre. Urban consultants receiving this patient are generally experienced and, therefore, confident placing a chest tube in their context. They have the equipment and personnel to do so. However, the consultant needs to understand the remote practitioner’s context, in which the procedure is rarely done and equipment may be lacking or out of date. Lack of practice in remote locations cause skills and confidence to decay, and equipment is seldom stocked for infrequently performed procedures. Such contextual differences must be effectively communicated between the two groups in order to establish mutual understanding and optimize patient care.

## Conclusions

Delivering in situ simulation to colleagues in rural and remote areas can present unique challenges but has a number of potential advantages, particularly with respect to high-stakes low-frequency procedures and communications. Telesimulation can teach these skills using simple, cost-effective, and commonly available technology. However, planning and data capture methods differ from face-to-face settings. Debriefing, the most important part of the simulation, must be done with particular care and respect in order to produce a sense of alliance between learners and mentors at distance. Therefore, faculty development specific to in situ simulation using technologies described herein may be required. This technical report offers an approach to overcome barriers and effectively deliver such education to remotely placed learners.
